# Alcohol pharmacotherapy dispensing trends in Australia between 2006 and 2023

**DOI:** 10.1093/alcalc/agae063

**Published:** 2024-09-06

**Authors:** Ebony Quintrell, Amy Page, Caitlin Wyrwoll, Alexander Larcombe, David B Preen, Osvaldo Almeida, Christopher Etherton-Beer, Erin Kelty

**Affiliations:** School of Population and Global Health, University of Western Australia, Nedlands 6009, Western Australia, Australia; Respiratory Environmental Health, Wal-Yan Respiratory Research Centre, The Kids Research Institute Australia, Nedlands 6009, Western Australia, Australia; School of Allied Health, University of Western Australia, Crawley 6009, Western Australia, Australia; The Kids Research Institute Australia, Nedlands 6009, Western Australia, Australia; School of Human Sciences, University of Western Australia, Crawley 6009, Western Australia, Australia; Respiratory Environmental Health, Wal-Yan Respiratory Research Centre, The Kids Research Institute Australia, Nedlands 6009, Western Australia, Australia; Occupation, Environment and Safety, School of Population Health, Curtin University, Bentley 6102, Western Australia, Australia; School of Population and Global Health, University of Western Australia, Nedlands 6009, Western Australia, Australia; Medical School, University of Western Australia, Nedlands 6009, Western Australia, Australia; Medical School, University of Western Australia, Nedlands 6009, Western Australia, Australia; School of Population and Global Health, University of Western Australia, Nedlands 6009, Western Australia, Australia

**Keywords:** naltrexone, acamprosate, alcohol use disorders, pharmacotherapy, prescription

## Abstract

**Aims:**

This study aimed to investigate acamprosate and naltrexone dispensing patterns in Australia.

**Methods:**

A 10% representative sample of medications subsidized by the Australian Pharmaceutical Benefits Scheme (PBS) was used to identify individuals who were dispensed naltrexone or acamprosate between January 2006 and December 2023. Data were used to examine concurrent dispensing, medication switching and treatment episode length, as well as changes in prevalence and incidence over time.

**Results:**

During the study, we identified 22 745 individuals with a total of 117 548 dispensed prescriptions (45.3% naltrexone, 43.0% acamprosate, and 11.7% concurrent dispensing). Alcohol pharmacotherapy dispensing occurred in 1354 per 100 000 individuals. It is estimated that 2.9% of individuals with an alcohol use disorder in Australia are receiving a PBS-listed pharmacological treatment. For both pharmacotherapies, individuals were most likely to be male (60.0%) and 35–54 years of age (56.0%). Individuals were more likely to switch from acamprosate to naltrexone rather than the reverse. From 2006 and 2023, the number of prevalent individuals treated with an alcohol pharmacotherapy significantly increased, driven mainly the use of naltrexone, which more than doubled over the study period. Incident naltrexone-treated individuals were more likely to remain on treatment for the recommended minimum 3-month period compared to acamprosate treated individuals, although overall dispensing for at least 3 months was low (5.1%).

**Conclusions:**

In Australia between 2006 and 2023, rates of naltrexone dispensing have substantially increased, while acamprosate dispensing showed minimal changes. However, the use of alcohol pharmacotherapies remains low compared with the likely prevalence of alcohol use disorders.

## Teaser

**Short Summary:** This study examined dispensing patterns of individuals prescribed alcohol pharmacotherapies in Australia from 2006 to 2023. Analysis revealed a significant rise in naltrexone dispensing, but minimal changes in acamprosate dispensing. Adherence to treatment for the recommended minimum 3-month period was low.

## Introduction

Globally, alcohol use disorders (AUD) are the most common substance use disorder (Degenhardt et al. 2018) with a mean lifetime prevalence of 8.6% (Glantz et al. 2020). The World Health Organisation (WHO) estimates the harmful use of alcohol is responsible for 4.6% of the global burden of disease, and 2.6 million deaths annually worldwide (World Health Organisation 2024). In Australia, AUD are highly prevalent, affecting an estimated 4.4% of individuals over 15 years (WHO 2018), with the harmful effects of alcohol costing Australia $66.8 billion per year (Whetton et al. 2021). Moreover, AUD can cause profound disruptions to relationships, work performance, and psychological wellbeing, as well as cause significant personal financial burdens (Grant et al. 2015). With such personal and societal costs associated with AUD, it is important to ensure individuals with the condition have access to effective treatments.

Both psychosocial and pharmacological options are available to manage AUD. Residential rehabilitation and psychosocial therapies are generally recommended in combination with pharmacotherapies for optimal effectiveness (Haber et al. 2021, Ray et al. 2019), which in Australia include naltrexone, acamprosate, and disulfiram (Haber et al. 2021). Of these, naltrexone and acamprosate are currently considered first-line treatments and are publicly subsidized by the Australian Pharmaceutical Benefit Scheme (PBS). Naltrexone is beneficial for reducing alcohol consumption, whereas acamprosate is beneficial for relapse prevention (McPheeters et al. 2023, MacKillop et al. 2022).

Despite the availability of alcohol pharmacotherapies, many individuals with AUD remain untreated. In Australia, only 1 in 3 people with AUD are thought to receive specific treatment during their lifetime and, among those who do seek treatment, contact may be delayed by a median 18 years after symptom onset (Chapman et al. 2015). Delayed treatment may be of particular concern when it comes to the use of alcohol pharmacotherapies, which have historically been underutilized (Manca et al. 2024, Månsson et al. 2024, Morley et al. 2016, Rittenberg et al. 2020) despite the efficacy (Jonas et al. 2014) and cost-effectiveness of such medications (Higginbotham et al. 2024). Existing research by a previous Australian prescription-based study estimated based on data from 2011 to 2012, that only 3% of people with AUD were receiving pharmacotherapy treatment in Australia (Morley et al. 2016). Moreover, of the individuals pharmacologically treated for AUD in Australia between 2009 and 2013, only 15–25% received treatment for the recommended minimum three month period (Morley et al. 2016).

It is important to revisit Australian data to identify trends in the uptake of alcohol pharmacotherapies since 2013, building upon the snapshot provided by Morley et al. (2016). This research aims to (i) examine temporal trends in prevalent individuals dispensed PBS-listed alcohol pharmacotherapies in Australia between 2006 and 2023, overall and by sex, and age, (ii) examine temporal trends in incident individuals dispensed an alcohol pharmacotherapy between 2013 and 2023, and (iii) identify treatment characteristics of incident alcohol pharmacotherapy individuals.

## Materials and methods

### Setting

This retrospective cohort study utilized a representative 10% sample of PBS data. The PBS is a program in which the Australian government subsidizes the cost of prescription medication for Australian residents (Department of Health and Aged Care 2024). Supplied by Services Australia, the PBS sample contained data from January 2006 to December 2023 and reflects all PBS claiming activity for approximately 10% of the eligible Australian population. The dataset includes demographic information, specifically sex, year of birth, and year of death in addition to a range of dispensing information (see Section Treatment exposures).

### Treatment exposures

During the study period, two alcohol pharmacotherapies were available for subsidy on the PBS: naltrexone (PBS Item code: 8370 M) and acamprosate (PBS Item code: 8357 W). Both naltrexone and acamprosate were priced above the PBS co-payment threshold for the entire study period, thus all general beneficiary and concession dispensing in the sample were captured. Naltrexone and acamprosate are exclusively listed on the PBS for the treatment of AUD, therefore limiting the dispensing of these medications for the treatment of other conditions. However, naltrexone is also used to treat opioid use disorders. Therefore, although discouraged, some clinicians may prescribe naltrexone under the PBS for the treatment of opioid use disorders.

For naltrexone, it is recommended individuals take one 50 mg oral tablet per day (Australian Medicines Handbook 2024). For individuals weighing 60 kg or more, the recommended dosage of acamprosate is two tablets taken three times daily (a total daily dose of 1998 mg) (Australian Medicines Handbook 2024). For individuals weighing less than 60 kg, it is recommended to take four acamprosate tablets per day split into three doses (total daily dose of 1332 mg) (Australian Medicines Handbook 2024). For calculations of exposure, the higher dose was used, as neither patient weight nor prescribed daily dose is included in the PBS 10% sample. National treatment guidelines recommend naltrexone and acamprosate are taken for at least 3–6 months, with treatment thereafter assessed on a case by case basis (Morley et al. 2021).

### Study cohort

The study included two cohorts of patients treated with acamprosate and/or naltrexone. The first included all individuals in the PBS 10% sample, aged 18 years and over at age of first AUD pharmacotherapy dispensing, and with a dispensing claim for acamprosate or naltrexone from January 2006 to December 2023. This cohort was termed the prevalence cohort.

The second cohort included individuals with a first alcohol pharmacotherapy dispensed between 2013 and 2023. Individuals with earlier dispensing (looking back to 2006) were excluded to remove any potential prevalent pool effect. This cohort was termed the incident cohort and was used to examine new users.

In both cohorts we also excluded individuals dispensed less than 120 tablets in total for acamprosate and 30 tablets in total for naltrexone (*n* = 51), as this is considered insufficient (<30 days’ worth of medication) for the treatment of AUD.

### Outcomes

Outcomes in this study included annual prevalent (using the prevalence cohort) and annual incident (using the incidence cohort) individuals dispensed either naltrexone or acamprosate. We estimated prevalence by identifying individuals with at least one alcohol pharmacotherapy dispensing within a given calendar year. Annual incidence was calculated as the number of new individuals with an alcohol pharmacotherapy dispensing within a given calendar year with the index (incident) dispensing denoted as the first original dispensing episode of either naltrexone or acamprosate that appeared in the data set for an individual person (with look back to 2006). For both, the denominator values utilized the adult (>18 years) Australian national population for that year (Australian Bureau of Statistics 2023) and were modified to 10% to correspond with the 10% PBS data sample. Incidence and prevalence are expressed as a crude rate (the proportion of individuals per 100 000 persons per year). The estimated end date of a medication supply was calculated as the quantity dispensed divided by the recommended daily dose (naltrexone = 1 x 50 mg tablet/day, acamprosate = 6 x 333 mg tablets/day) to determine an estimated medication supply length (days) from the dispensing date (Australian Medicines Handbook 2024). A treatment period was defined as the period between the index dispensing and date of the last dispensing plus the medication supply length and an additional 5-day window for prescription refill.

The percentage of individuals with a second and third dispensing episode was calculated from the index dispensing date. This was defined as a dispensing of the same medication within 5 days from the estimated end date of medication supply from the previous dispensing episode. Therefore, a gap of 5 days between the end of a dispensing episode and the beginning of the next dispensing episode was allowed (35 days since supply date for a standard dose). Concurrent use was defined as a dispensing for one alcohol pharmacotherapy within the supply period of the alternative alcohol pharmacotherapy. A second and third dispensing for an individual concurrently using both pharmacotherapies was defined as a dispensing of both naltrexone and acamprosate within the time-period. Median days on treatment was calculated as the number of days between the index supply date and the estimated end date of the last dispensing episode in that treatment period.

Switching from naltrexone to acamprosate or vice versa was defined as the absence of a refill dispensed for naltrexone or acamprosate and a dispensing of the alternative pharmacotherapy after the estimated end date of the last dispense plus a 5-day window (35 days) if the next dispensing occurred with a treatment gap of no more than 105 days (three-times the usual supply period). Where individuals switched multiple times, only their first switch was considered.

### Statistical analyses

We calculated annual prevalent and incident dispensing trends stratified by sex and age using Australian Bureau of Statistics population data (Australian Bureau of Statistics 2023). Characteristics of sex and age were presented using descriptive statistics. Data cleaning and statistical analysis were conducted using Stata/SE (version 17.0). Comparisons between two dichotomous categorical variables (e.g. percentage of individuals with a 3-month supply) were conducted using univariable logistic regressions. Trend graphs were produced using a combination of Joinpoint Regression Program (National Cancer Institute 2024) and Graphpad Prism (version 10.1.2; December 2023).

#### Joinpoint regression analysis

Temporal trends were examined with joinpoint regressions using Joinpoint Regression Program (Kim et al. 2022, National Cancer Institute 2024). Each Joinpoint represents a statistically significant change in trend. We used a log-linear model with a maximum of three joinpoints, whereby a weighted Bayesian information criterion model was used for model selection. Confidence intervals (CI) were calculated using the empirical quantile method. Annual percent change (APC) with 95% CIs was computed for each trend line segment. Average annual percentage change (AAPC) was also calculated for the whole time period (which assumes only one line with no joinpoints).

### Ethics

This study was approved by Services Australia and the University of Western Australia Human Research Ethics Committee (2022/ET000372).

## Results

### Prevalence cohort

In the 10% PBS sample from 2006 to 2023, we identified a total of 22 745 individuals and 117 548 dispensed alcohol pharmacotherapy prescriptions. Dispensing was similar among the two alcohol pharmacotherapies examined, with naltrexone accounting for 45.3% (*n* = 53 242) of dispensed prescriptions and acamprosate accounting for 43.0% (*n* = 50 528). Additionally, 11.7% (*n* = 13 778) of dispensing were classified as concurrent use of both acamprosate and naltrexone. Individuals dispensed an alcohol pharmacotherapy were predominantly male (60%) and 35–54 years of age (56%) (Table 1). From 2006 to 2023, 0.5% (*n* = 113) of individuals died in the same year as their initial dispensing.

**Table 1:** Number of individuals with at least one dispensing for an alcohol pharmacotherapy in Australia between January 2006 and December 2023, by sex and age.

	All APs (*n* = 21 909) *n* (%)	Naltrexone (*n* = 9470) *n* (%)	Acamprosate (*n* = 12 439) *n* (%)	Concurrent use (*n* = 836) *n* (%)
Sex				
Male	13,192 (60.2%)	5602 (59.2%)	7590 (61.0%)	490 (58.6%)
Age				
18–24	908 (4.1%)	469 (5.0%)	439 (3.5%)	35 (4.2%)
25–34	4135 (18.9%)	1854 (19.6%)	2281 (18.3%)	168 (20.1%)
35–44	6505 (29.7%)	2863 (30.2%)	3642 (29.3%)	260 (31.1%)
45–54	5739 (26.2%)	2392 (25.3%)	3347 (26.9%)	239 (28.6%)
55–64	3221 (14.7%)	1303 (13.8%)	1918 (15.4%)	101 (12.1%)
65+	1401 (6.4%)	589 (6.2%)	812 (6.5%)	33 (4.0%)

APs = Alcohol pharmacotherapies (excluding co-dispensing). Results are at the time of the individual’s index dispense (first dispense) that appeared in the dataset. ‘All APs’, ‘Naltrexone’ and ‘Acamprosate’ exclude concurrent use dispensing. Percentages may not add to exactly 100% as a result of rounding.

#### Concurrent dispensing and switching

In total, 8.7% (*n* = 1969) of individuals were concurrently dispensed both acamprosate and naltrexone. Over the study period, 0.8% (*n* = 183) of individuals switched from one pharmacotherapy to the alternative. Individuals were more likely to switch from acamprosate to naltrexone (*n* = 104) than from naltrexone to acamprosate (*n* = 79) (OR = 1.77; 95% CI: 1.32–2.37).

#### Temporal trends in prevalence of alcohol pharmacotherapy dispensing by sex and age

**Figure 1:**
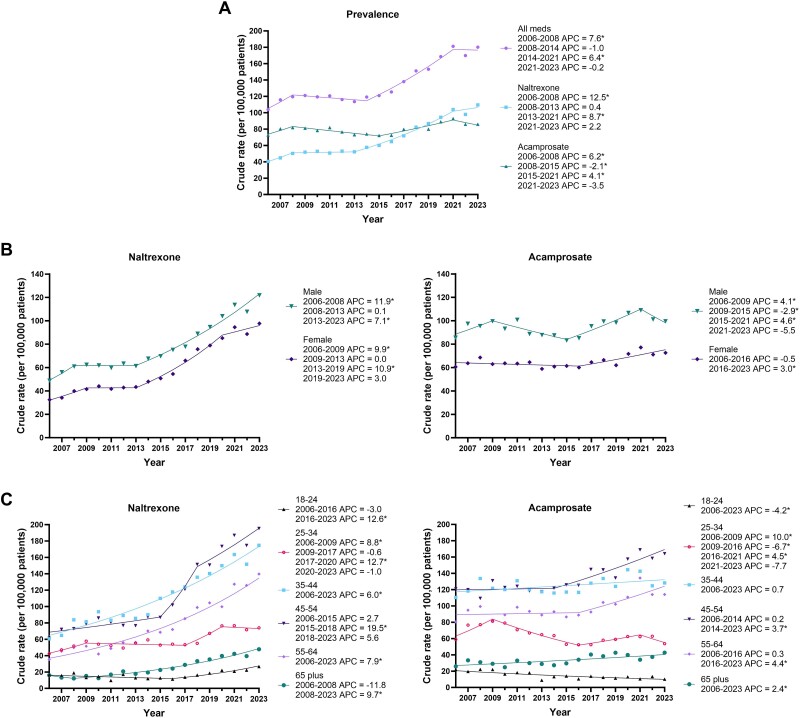
(A) Temporal trends in prevalent Australian adults dispensed an alcohol pharmacotherapy (2006–2023) by (B) sex, (C) age group. All meds = Both naltrexone and acamprosate pharmacotherapy dispensing data combined. ^*^ indicates the APC is significant different from zero at the alpha = 0.05 level

Between 2006 and 2023, the number of prevalent individuals dispensed an alcohol pharmacotherapy increased 1.7-fold from 1039 to 1801 per 100 000 individuals per year (AAPC = 3.1%; 95% CI: 2.4–3.8). Prevalent individual numbers plateaued between 2008 and 2014, followed by a significant 1.5-fold increase until 2021 (Fig. 1A; eTable 1). The number of prevalent naltrexone treated individuals increased 2.6-fold over the study period (AAPC = 5.9%; 95% CI: 5.3–6.5), whereas the number of individuals treated with acamprosate increased only modestly from 2006 to 2023 (AAPC = 0.8%; 95% CI: 0.2–1.4). This general pattern remained when delineated by sex, although a sharp, but statistically non-significant decline in male acamprosate treated individuals from 2021 to 2023 should be noted (Fig. 1B; eTables 2 and 3). There were consistently more male individuals than female throughout the study period.

In terms of age, the crude rate of pharmacotherapy use was highest in individuals aged 35–44 and 45–54 years for both naltrexone and acamprosate treated individuals (Fig. 1C; eTables 4 and 5). From 2006 to 2023, all age group crude rates increased apart from acamprosate dispensed individuals in the 18–24 and 25–34 age categories which reduced by 51% and 13% respectively. For naltrexone treated individuals, the 45–54 age category had a 2.2-fold increase from 2015 to 2023. A similar but less steep, 1.4-fold increase was also seen in this age category for acamprosate treated individuals from 2014 to 2023.

### Incidence cohort

#### Temporal trends in incidence of alcohol pharmacotherapy dispensing by sex and age

A total of 14 302 individuals were identified as having an incident alcohol pharmacotherapy dispensed in Australia from 1 January 2013, to 31 December 2023. In this 11-year time period, 58 864 alcohol pharmacotherapy prescriptions were dispensed. For incident individuals, naltrexone accounted for 51.1% (*n* = 30 100) of dispensed prescriptions compared to 39.4% (*n* = 23 163) for acamprosate, while 9.5% (*n* = 5601) of dispensing were classified as concurrent use. The mean age at first dispensing was 44 ± 12.6 years (range 18–90) for individuals treated with naltrexone, 45 ± 12.4 years (range 18–91) for acamprosate, and 44 ± 11.9 (range 19–78) for concurrently dispensed individuals.

**Figure 2:**
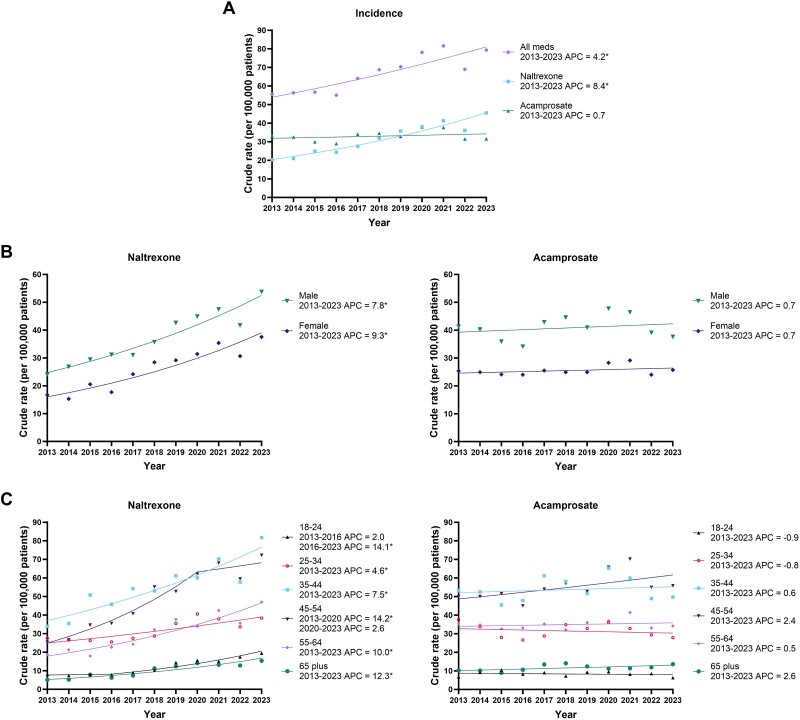
(A) Temporal trends in incident Australian adults dispensed an alcohol pharmacotherapy (2013–2023) by (B) sex, and (C) age group. All meds = Both naltrexone and acamprosate pharmacotherapy 1dispensing data combined. ^*^ indicates the APC is significantly different from zero at the alpha = 0.05 level. Individuals with concurrent use as their incident dispensing were excluded from estimates of naltrexone and acamprosate incidence

The number of incident patients dispensed an alcohol pharmacotherapy significantly increased 1.5-fold over the 11-year period (APC: 4.2%; 95% CI: 2.7–5.6; Fig. 2A; eTable 6). This increase was primarily driven by naltrexone which more than doubled (APC: 8.4%; 95% CI: 6.2–10.7), with no significant change in the annual incidence of acamprosate dispensing (APC: .7%; 95% CI: −2.8 to 4.4). These patterns remained when delineated by sex (Fig. 2B; eTables 7 and 8).

Increases in the incidence of naltrexone dispensing were observed for all age groups, with the largest growth in the 45–54 age group with a 2.7-fold increase, and 65 plus age group with a 3.2-fold increase. For incident acamprosate individuals, no age groups had any statistically significant changes over the study period (Fig. 2D; eTables 9 and 10).

#### Treatment episode characteristics

##### Comparing naltrexone to acamprosate

Overall, 78.7% (*n* = 10 897) of individuals completed only 1 month of treatment for their incident treatment episode, while 5.0% (*n* = 693) completed the 3-month recommended treatment period. The majority of incident individuals (51%, *n* = 7055) were dispensed acamprosate for their incident treatment episode rather than naltrexone (Table 2). However, overall, there were more naltrexone dispensings during the study period compared with acamprosate. Incident naltrexone treated individuals remained on treatment for a 15% longer duration than acamprosate treated individuals for their incident treatment episode. Compared to acamprosate treated individuals, naltrexone treated individuals were more likely to return for a second and third dispensing in their incident treatment episode and were 89% more likely to remain on treatment for the recommended 3-month time-period.

**Table 2:** Treatment patterns of alcohol pharmacotherapy dispensing in Australia between January 2013 and December 2023.

Incident episode	Acamprosate	Naltrexone	Effect measure (95% CI)
Individuals, *n* (%)	7055 (51.0%)	6787 (49.0%)	
Dispensing, *n* (%)	8833 (47.2%)	9866 (52.8%)	
Median days of incident treatment episode (IQR)	30 (30, 30)	30 (30, 46)	IRR = 1.15 (1.13–1.16)^a^
Second dispense, *n* (%)	1074 (15.2%)	1871 (27.6%)	OR = 2.12 (1.95–2.31)^a^
Third dispense, *n* (%)	349 (5.0%)	599 (8.8%)	OR = 1.86 (1.62–2.13)^a^
Individuals with ≥3-month incident treatment length, *n* (%)	251 (3.6%)	442 (6.5%)	OR = 1.89 (1.61–2.21)^a^

OR = odds ratio for naltrexone compared to acamprosate; IRR = incidence rate ratio; CI = confidence interval; IQR = interquartile range; excludes concurrent use dispensing. ^a^*P* < 0.001.

##### Comparing single dispensing to concurrent dispensing

Concurrent dispensing of acamprosate and naltrexone was associated with a marginal but significant (18%) increase in the median length of incident treatment episode compared with single dispensing (Table 3). However, concurrent dispensing was not associated with an increase in the percentage of individuals receiving a second or third prescription, nor the percentage of individuals remaining on treatment for 3-months. Regardless of concurrent or single dispensing, only 5.1% (*n* = 732) of individuals remained on treatment for the recommended 3-months for their incident treatment episode.

**Table 3:** Patterns of dispensing in individuals who were dispensed either a single alcohol pharmacotherapy or were concurrently dispensed two different alcohol pharmacotherapies between January 2013 and December 2023.

Incident episode	Single-dispense	Concurrent use	Effect measure (95% CI)
Individuals, *n* (%)	13 842 (96.8%)	460 (3.2%)	
Dispensing, *n* (%)	18 699 (93.8%)	1235 (6.2%)	
Median days of incident treatment episode (IQR)	30 (30, 30)	35 (30, 54)	IRR = 1.18 (1.13–1.23)^a^
Second dispense, *n* (%)	2945 (21.3%)	102 (22.2%)	OR = 1.05 (0.84–1.32)
Third dispense, *n* (%)	948 (6.9%)	32 (7.0.%)	OR = 1.02 (.71–1.47)
Individuals with ≥3-month incident treatment length, *n* (%)	693 (5.0%)	30 (6.5%)	OR = 1.32 (.91–1.93)

Single-dispense: A single dispense of either acamprosate or naltrexone; OR = odds ratio for single dispensing compared to concurrent use dispensing; IRR = incidence rate ratio; CI = confidence interval. ^a^*P* < 0.001.

## Discussion

The dispensing of PBS subsidized alcohol pharmacotherapies has increased in Australia between 2006 and 2023. This growth was attributed to an increased number of naltrexone treated individuals, which more than doubled over the study period. In contrast, only modest increases were seen in the number of acamprosate treated individuals. With both naltrexone and acamprosate showing similar efficacy (Jonas et al. 2014) and side-effect tolerability (Haber et al. 2021), the difference in therapeutic use may be due to the different treatment effects of the medications. Evidence suggests that naltrexone may be more efficacious in reducing heavy drinking and craving, while acamprosate has stronger efficacy in promoting and maintaining abstinence (McPheeters et al. 2023, MacKillop et al. 2022, Haber et al. 2021). Thus, we may be seeing a shift in treatment goals, where individuals may be aiming to reduce their drinking rather than completely abstain. Alternatively, naltrexone might present a more manageable treatment option, as individuals only require one tablet per day, as opposed to acamprosate’s six tablets per day.

Trends in the prevalence of AUD in Australia are difficult to ascertain due to infrequent measurement. The two earliest Australian studies show a decline from 6.5% in 1997 to 4.3% in 2007 (Teesson et al. 2000, Teesson et al. 2010), while the most recent estimate from 2016 reports a prevalence of 4.4% (WHO 2018). In comparison, only approximately 0.13% of people in Australia are receiving a PBS listed alcohol pharmacotherapy, equating to ~2.9% of individuals with AUD receiving an alcohol pharmacotherapy. This reflects the results of a previous Australian prescription-based study which estimated 2.7–3% of individuals with AUD in Australia were prescribed an alcohol pharmacotherapy (Morley et al. 2016). The reasons for this treatment gap may be attributed to the many perceived barriers facing people with AUD. Prominent impediments to treatment may be patient-based (stigma, poor perception of treatment need), physician-based (lack of knowledge, reluctance to prescribe pharmacotherapy), medicine-based (adverse effects, limited efficacy, inconvenient dosing schedule), or system-based (lack of treatment services, lack of advocacy) (Haber and Morley 2016, Finn et al. 2023). Moreover, the heterogeneity of AUD means that alcohol pharmacotherapies do not work for every individual (Heilig et al. 2019), with a meta-analysis reporting the number needed to treat for both naltrexone and acamprosate is 12 (Jonas et al. 2014). While the increase in alcohol pharmacotherapy dispensing our study reported indicates that more people are being treated over time, the proportion of people with AUD receiving such treatment likely remains low. Additionally, due to the notable gaps in Australian data on the prevalence of AUD, it is unclear if the prevalence of AUD has also increased over the study period or if more individuals are seeking pharmacotherapy treatment.

The demographics of individuals in this study were similar to Morley et al. (2016), where male individuals outnumbered female individuals by 20%. This predominance of male individuals reflects the higher prevalence of AUD among males in Australia and throughout the world (WHO 2018). Based on Australian data in 2016, 6.1% of males are estimated to have AUD compared with 2.7% of females (WHO 2018). This equates to approximately 0.15% of males and 0.12% of females receiving a PBS listed alcohol pharmacotherapy. While male individuals far outnumber female individuals for diagnoses of AUD, there is a smaller scale difference between the two sexes for alcohol pharmacotherapy prescriptions. This may suggest a bigger treatment gap among males with AUD compared to females. Reflecting this issue, a study conducted in the USA reported female individuals were more likely (10.9%) to use medication for the treatment of their AUD compared to males (6.5%) (Hu et al. 2022).

Irrespective of sex, the highest proportion of individuals with an alcohol pharmacotherapy dispensing were in the 35–44 year age category, which is consistent with previously published Australian data from 2009 to 2013 (Morley et al. 2016). For all incident pharmacotherapy individuals, the median age at first dispensing was 45 years. In comparison, the median age of AUD symptom onset in Australia is estimated to be 19 years (Solmi et al. 2022), which could suggest an estimated 26-year treatment delay. This is consistent with research by Chapman et al. (2015) who observed a median treatment delay of 18 years among Australians with AUD.

Naltrexone treated individuals were more likely to return for a repeat dispensing and were also more likely to remain on treatment for the recommended 3-month time period in their incident treatment episode compared with those receiving acamprosate. This finding suggests better adherence of naltrexone compared to acamprosate, reflecting the results of Morley et al. 2016. The reason for the lower adherence to acamprosate may be partially attributed to the dosage regimen. Acamprosate treatment involves taking 2 tablets three times per day (a total of 6 tablets per day) which may make adherence more challenging compared to medications such as naltrexone which only require 1 tablet per day (Koeter et al. 2010). Minimizing the frequency and complexity of a dosage regimen has been shown to improve medication adherence (Osterberg and Blaschke 2005, Weiss 2004). For acamprosate, it is suggested adherence for prolonged periods should be specifically supported due to the pill burden and more complex dosage regimen (Haber et al. 2021). However, despite pharmacotherapy type or concurrent dispensing, only 5.1% of incident individuals remained on treatment for the recommended 3-month period, displaying even lower adherence than previously found in Australia from 2009 to 2013 (Morley et al. 2016).

There has been some evidence to suggest acamprosate and naltrexone in combination produces enhanced relapse prevention, with each treatment working via different pathways (Feeney et al. 2006, Kiefer et al. 2003, Kiefer and Wiedemann 2004). However, two meta-analyses from 2010 found that both pharmacotherapies in combination did not significantly decrease the risk of returning to any or heavy alcohol consumption compared to either pharmacotherapy alone or placebo (Rosner et al. 2010a, Rosner et al. 2010b). In the current dataset, individuals with an incident concurrent dispensing had a marginally longer incident episode compared to single dispensed individuals, however, this was not found for repeat dispensing, or the percentage of individuals treated for ≥3 months. Although, concurrent dispensing was not commonly used in Australia, only constituting 11.7% of total alcohol-related dispensings in the data. Additionally, concurrent dispensing may have been used in more complex cases, resulting in lower adherence.

Individuals were more likely to switch from acamprosate to naltrexone than from naltrexone to acamprosate, consistent with findings from Morley et al. (2016). These findings were similar despite the methodological differences between the studies. Morley et al. (2016) calculated switching as a switch in an individual’s lifetime, meaning even if an individual had discontinued treatment for a number of years before recommencing treatment with the alternative pharmacotherapy, it was considered a switch. In this study, however, switching was defined as changing to the alternative medication within 105 days of the last supply date of the original medication. Nevertheless, more individuals switching from acamprosate to naltrexone could be attributed to the six tablets per day treatment regimen of acamprosate, compared to the 1 tablet per day needed for naltrexone.

### Limitations

There are a few limitations inherent in using the PBS dataset for research purposes. The PBS is based on dispensing data with no information on individual direction for use or actual usage. This was particularly a limitation for acamprosate, where two doses are typically dispensed based on patient weight. An additional factor to consider is that the PBS dataset does not capture private dispensing or dispensing in in-patients within public hospitals. Since the 10% PBS sample only includes the year of death as opposed to the exact death date, it was not possible to censor the supply period if the individual died. Finally, the interpretation of this dataset assumes all individuals being dispensed naltrexone and acamprosate are being treated for AUD as these medications are only listed on the PBS for this purpose. However, it is important to consider there is potential for clinicians to prescribe alcohol pharmacotherapies off-label for other medical conditions such as opioid use disorders while still using the PBS code. While expected to be minimal, it could mean some individuals in this dataset are not receiving alcohol pharmacotherapies for the purpose of treating AUD. Given these limitations, future research may endeavor to explore the use of other alcohol pharmacotherapies such as disulfiram, the time from AUD symptom onset to treatment contact, and estimates of the current prevalence of AUD in Australia.

### Conclusion

In Australia, the number of naltrexone treated individuals has substantially increased since 2006, while acamprosate dispensing has changed minimally. Regardless of pharmacotherapy type, adherence to treatment for the recommended minimum 3-month period remains concerningly low, at 5.1%. Additionally, the treatment of individuals with AUD also remains low, with estimates suggesting only 2.9% of people with AUD are receiving a PBS listed pharmacological treatment. However, it is unclear if the prevalence of AUD has also increased during this period or if more individuals are seeking pharmacotherapy treatment.

## Supplementary Material

Supplementary_Tables_agae063

## Data Availability

The 10% PBS database is available from Services Australia (https://www.servicesaustralia.gov.au) upon approved application.
