# Supplementary material for: Alcohol pharmacotherapy dispensing trends in Australia between 2006 and 2023
Source: Alcohol Alcohol. 2024 Sep 6;59(5):agae063. doi: 10.1093/alcalc/agae063 (PMC11379491; doi:10.1093/alcalc/agae063)
Supplement: Supplementary_Tables_agae063 [file supplementary_tables_agae063.docx]

**Supplementary Tables**

**Alcohol pharmacotherapy dispensing trends in Australia between 2006 and 2023**

Ebony Quintrell^1,2^, Amy Page^3^, Caitlin Wyrwoll^4,5^, Alexander Larcombe^2,6^, David B Preen^1^, Osvaldo Almeida^7^, Christopher Etherton-Beer^7^, Erin Kelty^1^

^1^ School of Population and Global Health, University of Western Australia, Nedlands, Western Australia.

^2^ Respiratory Environmental Health, Wal-yan Respiratory Research Centre, Telethon Kids Institute, Nedlands, Western Australia, Australia.

^3^ School of Allied Health, University of Western Australia, Nedlands, Western Australia

^4^ Telethon Kids Institute, Nedlands, Western Australia, Australia.

^5^ School of Human Sciences, University of Western Australia, Nedlands, Western Australia, Australia.

^6^ Occupation, Environment and Safety, School of Population Health, Curtin University, Perth, Western Australia.

^7^ Medical School, University of Western Australia, Nedlands, Western Australia

**Supplementary Tables**

**Prevalence Cohort**

**eTable 1.** Results of Joinpoint trend analysis for prevalent Australian adults dispensed an alcohol pharmacotherapy (2006 – 2023).

| **Cohort** | **Segment** | **Trend lower and upper end points** | **Annual Percent Change (APC)** | **95% Confidence Interval (CI)** | **P-Value** |
| --- | --- | --- | --- | --- | --- |
| All medications | 1 | 2006 – 2008 | 7.6* | 2.3, 14.4 | 0.005 |
|  | 2 | 2008 – 2014 | -1.0 | -5.6, 0.5 | 0.113 |
|  | 3 | 2014 – 2021 | 6.4* | 4.7, 11.6 | 0.001 |
|  | 4 | 2021 – 2023 | -0.2 | -6.1, 5.0 | 0.822 |
| Naltrexone | 1 | 2006 – 2008 | 12.5* | 6.9, 17.9 | < 0.001 |
|  | 2 | 2008 – 2013 | 0.4 | -3.0, 2.0 | 0.744 |
|  | 3 | 2013 – 2021 | 8.7* | 7.9, 12.2 | < 0.001 |
|  | 4 | 2021 – 2023 | 2.2 | -2.3, 6.9 | 0.228 |
| Acamprosate | 1 | 2006 – 2008 | 6.2* | 1.1, 11.8 | 0.006 |
|  | 2 | 2008 – 2015 | -2.1* | -5.8, -1.2 | 0.003 |
|  | 3 | 2015 – 2021 | 4.1* | 2.7, 8.3 | 0.007 |
|  | 4 | 2021 – 2023 | -3.5 | -8.6, 1.2 | 0.162 |
| **Cohort** | **Segment** | **Trend lower and upper end points** | **Average Annual Percent Change (AAPC)** | **95% Confidence Interval (CI)** | **P-Value** |
| Both | Full range | 2006 - 2023 | 3.1* | 2.4, 3.8 | < 0.001 |
| Naltrexone | Full range | 2006 - 2023 | 5.9* | 5.3, 6.5 | < 0.001 |
| Acamprosate | Full range | 2006 - 2023 | 0.8* | 0.2, 1.4 | 0.007 |

All medications = both naltrexone and acamprosate pharmacotherapy data combined.

* Indicates the APC or AAPC is significantly different from zero at the alpha = 0.05 level.

**eTable 2.** Temporal trends in prevalent Australian adults dispensed an alcohol pharmacotherapy (2006 – 2023) by sex for naltrexone.

| **Naltrexone cohort** | **Segment** | **Trend lower and upper end points** | **Annual Percent Change (APC)** | **95% Confidence Interval (CI)** | **P-Value** |
| --- | --- | --- | --- | --- | --- |
| Male | 1 | 2006 – 2008 | 11.9* | 5.6, 18.3 | < 0.001 |
|  | 2 | 2008 – 2013 | 0.1 | -4.1, 2.0 | 0.869 |
|  | 3 | 2013 – 2023 | 7.1* | 6.3, 8.4 | 0.002 |
| Female | 1 | 2006 – 2009 | 9.9* | 6.1, 18.4 | < 0.001 |
|  | 2 | 2009 – 2013 | 0.0 | -4.5, 3.4 | 0.956 |
|  | 3 | 2013 – 2020 | 10.9* | 9.5, 17.6 | < 0.001 |
|  | 4 | 2020 – 2023 | 3.0 | -5.4, 7.1 | 0.250 |
| **Naltrexone cohort** | **Segment** | **Trend lower and upper end points** | **Average Annual Percent Change (AAPC)** | **95% Confidence Interval (CI)** | **P-Value** |
| Male | Full Range | 2006 – 2023 | 5.5* | 5.1, 6.1 | < 0.001 |
| Female | Full Range | 2006 – 2023 | 6.7* | 5.8, 7.5 | < 0.001 |

* Indicates the APC or AAPC is significantly different from zero at the alpha = 0.05 level.

**eTable 3.** Temporal trends in prevalent Australian adults dispensed an alcohol pharmacotherapy (2006 – 2023) by sex for acamprosate.

| **Acamprosate cohort** | **Segment** | **Trend lower and upper end points** | **Annual Percent Change (APC)** | **95% Confidence Interval (CI)** | **P-Value** |
| --- | --- | --- | --- | --- | --- |
| Male | 1 | 2006 – 2009 | 4.1* | 0.5, 12.1 | 0.022 |
|  | 2 | 2009 – 2015 | -2.9* | -8, -1.3 | 0.007 |
|  | 3 | 2015 – 2021 | 4.6* | 3, 10.5 | 0.004 |
|  | 4 | 2021 – 2023 | -5.5 | -12.1, 0.5 | 0.076 |
| Female | 1 | 2006 – 2016 | -0.5 | -4.3, 0.5 | 0.313 |
|  | 2 | 2016 – 2023 | 3.0* | 1.3, 8.9 | 0.003 |
| **Acamprosate cohort** | **Segment** | **Trend lower and upper end points** | **Average Annual Percent Change (AAPC)** | **95% Confidence Interval (CI)** | **P-Value** |
| Male | Full Range | 2006 – 2023 | 0.6 | -0.1, 1.4 | 0.084 |
| Female | Full Range | 2006 – 2023 | 0.9* | 0.3, 1.6 | 0.008 |

* Indicates the APC or AAPC is significantly different from zero at the alpha = 0.05 level.

**eTable 4.** Temporal trends in prevalent Australian adults dispensed an alcohol pharmacotherapy (2006 – 2023) by age group for naltrexone.

| **Naltrexone cohort** | **Segment** | **Trend lower and upper end points** | **Annual Percent Change (APC)** | **95% Confidence Interval (CI)** | **P-Value** |
| --- | --- | --- | --- | --- | --- |
| 18-24 | 1 | 2006 – 2016 | -3.0 | -10.7, 0.4 | 0.087 |
|  | 2 | 2016 – 2023 | 12.6* | 6.0, 31.8 | < 0.001 |
| 25-34 | 1 | 2006 – 2009 | 8.8* | 3.6, 17.0 | < 0.001 |
|  | 2 | 2009 – 2017 | -0.6 | -4.5, 0.5 | 0.233 |
|  | 3 | 2017 – 2020 | 12.7* | 7.0, 16.4 | 0.005 |
|  | 4 | 2020 – 2023 | -1.0 | -8.0, 3.0 | 0.471 |
| 35-44 | 1 | 2006 – 2023 | 6.0* | 5.1, 6.9 | < 0.001 |
| 45-54 | 1 | 2006 – 2015 | 2.7 | -0.2, 4.6 | 0.061 |
|  | 2 | 2015 – 2018 | 19.5* | 6.5, 24.3 | 0.027 |
|  | 3 | 2018 – 2023 | 5.6 | -2.5, 9.7 | 0.093 |
| 55-64 | 1 | 2006 – 2023 | 7.9* | 6.7, 9.3 | < 0.001 |
| 65+ | 1 | 2006 – 2008 | -11.8 | -19.6, 7.6 | 0.276 |
|  | 2 | 2008 – 2023 | 9.7* | 8.3, 14.1 | 0.014 |
| **Naltrexone cohort** | **Segment** | **Trend lower and upper end points** | **Average Annual Percent Change (AAPC)** | **95% Confidence Interval (CI)** | **P-Value** |
| 18-24 | Full Range | 2006 – 2023 | 3.2* | 1.0, 5.2 | 0.006 |
| 25-34 | Full Range | 2006 – 2023 | 3.2* | 2.4, 3.9 | < 0.001 |
| 35-44 | Full Range | 2006 – 2023 | 6.0* | 5.1, 6.9 | < 0.001 |
| 45-54 | Full Range | 2006 – 2023 | 6.4* | 5.3, 7.1 | < 0.001 |
| 55-64 | Full Range | 2006 – 2023 | 7.9* | 6.7, 9.3 | < 0.001 |
| 65+ | Full Range | 2006 – 2023 | 6.9* | 5.8, 9.1 | < 0.001 |

* Indicates the APC or AAPC is significantly different from zero at the alpha = 0.05 level.

**eTable 5.** Temporal trends in prevalent Australian adults dispensed an alcohol pharmacotherapy (2006 – 2023) by age group for acamprosate.

| **Acamprosate cohort** | **Segment** | **Trend lower and upper end points** | **Annual Percent Change (APC)** | **95% Confidence Interval (CI)** | **P-Value** |
| --- | --- | --- | --- | --- | --- |
| 18-24 | 1 | 2006 – 2023 | -4.2* | -6.1, -2.1 | < 0.001 |
| 25-34 | 1 | 2006 – 2009 | 10.0* | 3.9, 19.5 | 0.001 |
|  | 2 | 2009 – 2016 | -6.7* | -11.8, -4.8 | 0.001 |
|  | 3 | 2016 – 2021 | 4.5 | -1.7, 12.0 | 0.062 |
|  | 4 | 2021 – 2023 | -7.7 | -16.2, 1.1 | 0.069 |
| 35-44 | 1 | 2006 – 2023 | 0.7 | -0.1, 1.5 | 0.087 |
| 45-54 | 1 | 2006 – 2014 | 0.2 | -5.6, 1.8 | 0.991 |
|  | 2 | 2014 – 2023 | 3.7* | 2.4, 9.4 | 0.009 |
| 55-64 | 1 | 2006 – 2016 | 0.3 | -7.7, 2.4 | 0.968 |
|  | 2 | 2016 – 2023 | 4.4* | 1.3, 15.2 | 0.035 |
| 65+ | 1 | 2006 – 2023 | 2.4* | 0.9, 4.0 | 0.003 |
| **Acamprosate cohort** | **Segment** | **Trend lower and upper end points** | **Average Annual Percent Change (AAPC)** | **95% Confidence Interval (CI)** | **P-Value** |
| 18-24 | Full Range | 2006 – 2023 | -4.2* | -6.1, -2.1 | < 0.001 |
| 25-34 | Full Range | 2006 – 2023 | -0.8 | -1.8, 0.2 | 0.092 |
| 35-44 | Full Range | 2006 – 2023 | 0.7 | -0.1, 1.5 | 0.087 |
| 45-54 | Full Range | 2006 – 2023 | 2.1* | 1.3, 2.8 | < 0.001 |
| 55-64 | Full Range | 2006 – 2023 | 2.0* | 0.8, 3.1 | 0.002 |
| 65+ | Full Range | 2006 – 2023 | 2.4* | 0.9, 4.0 | 0.003 |

* Indicates the APC or AAPC is significantly different from zero at the alpha = 0.05 level.

**Incidence Cohort**

**eTable 6.** Temporal trends in incident Australian adults dispensed an alcohol pharmacotherapy (2013 – 2023)

| **Cohort** | **Segment** | **Trend lower and upper end points** | **Annual Percent Change (APC)** | **95% Confidence Interval (CI)** | **P-Value** |
| --- | --- | --- | --- | --- | --- |
| All medications | 1 | 2013 – 2023 | 4.2* | 2.7, 5.6 | < 0.001 |
| Naltrexone | 1 | 2013 – 2023 | 8.4* | 6.2, 10.7 | < 0.001 |
| Acamprosate | 1 | 2013 – 2023 | 0.7 | -2.8, 4.4 | 0.594 |

All medications = both naltrexone and acamprosate pharmacotherapy data combined.

In the absence of a joinpoint, the AAPC is equivalent to the APC over the entire period.

* Indicates the APC is significantly different from zero at the alpha = 0.05 level.

**eTable 7.** Temporal trends in incident Australian adults dispensed an alcohol pharmacotherapy (2013 – 2023) by sex for naltrexone.

| **Naltrexone cohort** | **Segment** | **Trend lower and upper end points** | **Annual Percent Change (APC)** | **95% Confidence Interval (CI)** | **P-Value** |
| --- | --- | --- | --- | --- | --- |
| Male | 1 | 2013 – 2023 | 7.8* | 6.2, 9.5 | < 0.001 |
| Female | 1 | 2013 – 2023 | 9.3* | 7, 11.7 | < 0.001 |

In the absence of a joinpoint, the AAPC is equivalent to the APC over the entire period.

* Indicates the APC is significantly different from zero at the alpha = 0.05 level.

**eTable 8.** Temporal trends in incident Australian adults dispensed an alcohol pharmacotherapy (2013 – 2023) by sex for acamprosate.

| **Acamprosate cohort** | **Segment** | **Trend lower and upper end points** | **Annual Percent Change (APC)** | **95% Confidence Interval (CI)** | **P-Value** |
| --- | --- | --- | --- | --- | --- |
| Male | 1 | 2013 – 2023 | 0.7 | -2.7, 4.3 | 0.624 |
| Female | 1 | 2013 – 2023 | 0.7 | -0.8, 2.3 | 0.332 |

In the absence of a joinpoint, the AAPC is equivalent to the APC over the entire period.

* Indicates the APC is significantly different from zero at the alpha = 0.05 level.

**eTable 9.** Temporal trends in incident Australian adults dispensed an alcohol pharmacotherapy (2013 – 2023) by age for naltrexone.

| **Naltrexone cohort** | **Segment** | **Trend lower and upper end points** | **Annual Percent Change (APC)** | **95% Confidence Interval (CI)** | **P-Value** |
| --- | --- | --- | --- | --- | --- |
| 18-24 | 1 | 2013 – 2016 | 2.0 | -9.6, 9.1 | 0.545 |
|  | 2 | 2016 – 2023 | 14.1* | 11.9, 22.0 | < 0.001 |
| 25-34 | 1 | 2013 – 2023 | 4.6* | 1.1, 8.1 | 0.011 |
| 35-44 | 1 | 2013 – 2023 | 7.5* | 4.5, 10.7 | < 0.001 |
| 45-54 | 1 | 2013 – 2020 | 14.2* | 11.0, 30.9 | 0.001 |
|  | 2 | 2020 – 2023 | 2.6 | -12.2, 10.9 | 0.555 |
| 55-64 | 1 | 2013 – 2023 | 10.0* | 6.3, 14.0 | < 0.001 |
| 65+ | 1 | 2013 – 2023 | 12.3* | 6.4, 18.7 | < 0.001 |
| **Naltrexone cohort** | **Segment** | **Trend lower and upper end points** | **Average Annual Percent Change (AAPC)** | **95% Confidence Interval (CI)** | **P-Value** |
| 18-24 | Full Range | 2013 – 2023 | 10.3* | 8.2, 12.7 | < 0.001 |
| 25-34 | Full Range | 2013 – 2023 | 4.6* | 1.1, 8.1 | 0.011 |
| 35-44 | Full Range | 2013 – 2023 | 7.5* | 4.5, 10.7 | < 0.001 |
| 45-54 | Full Range | 2013 – 2023 | 10.6* | 7.7, 14.1 | < 0.001 |
| 55-64 | Full Range | 2013 – 2023 | 10.0* | 6.3, 14.0 | < 0.001 |
| 65+ | Full Range | 2013 – 2023 | 12.3* | 6.4, 18.7 | < 0.001 |

* Indicates the APC or AAPC is significantly different from zero at the alpha = 0.05 level.

**eTable 10.** Temporal trends in incident Australian adults dispensed an alcohol pharmacotherapy (2013 – 2023) by age for acamprosate.

| **Acamprosate Cohort** | **Segment** | **Trend lower and upper end points** | **Annual Percent Change (APC)** | **95% Confidence Interval (CI)** | **P-Value** |
| --- | --- | --- | --- | --- | --- |
| 18-24 | 1 | 2013 – 2023 | -0.9 | -4.1, 2.5 | 0.593 |
| 25-34 | 1 | 2013 – 2023 | -0.8 | -4.3, 3 | 0.650 |
| 35-44 | 1 | 2013 – 2023 | 0.6 | -3.6, 5 | 0.714 |
| 45-54 | 1 | 2013 – 2023 | 2.4 | -0.5, 5.3 | 0.094 |
| 55-64 | 1 | 2013 – 2023 | 0.5 | -1.7, 2.8 | 0.545 |
| 65+ | 1 | 2013 – 2023 | 2.6 | -0.3, 5.6 | 0.075 |

In the absence of a joinpoint, the AAPC is equivalent to the APC over the entire period.

* Indicates the APC is significantly different from zero at the alpha = 0.05 level.
